# Depression and Oral FTC/TDF Pre-exposure Prophylaxis (PrEP) Among Men and Transgender Women Who Have Sex With Men (MSM/TGW)

**DOI:** 10.1007/s10461-015-1082-2

**Published:** 2015-06-16

**Authors:** Patricia A. Defechereux, Megha Mehrotra, Albert Y. Liu, Vanessa M. McMahan, David V. Glidden, Kenneth H. Mayer, Lorena Vargas, K. Rivet Amico, Piotr Chodacki, Telmo Fernandez, Vivian I. Avelino-Silva, David Burns, Robert M. Grant, for the iPrEx Study Team

**Affiliations:** J. David Gladstone Institutes, Grant Lab, GIVI, 5th Floor, 1650 Owens Street, San Francisco, 94158 CA USA; Bridge HIV, San Francisco Department of Public Health, San Francisco, CA USA; University of California, San Francisco, CA USA; Fenway Community Health, Boston, MA USA; Investigaciones Medicas en Salud, Lima, Peru; School of Public Health, University of Michigan, Ann Arbor, MI USA; Desmond Tutu HIV Foundation, University of Cape Town, Cape Town, South Africa; Fundacion Ecuatoriana Equidad, Guayaquil, Ecuador; University of Sao Paulo Medical School, Sao Paulo, Brazil; National Institutes of Health, Bethesda, MD USA; San Francisco AIDS Foundation, San Francisco, CA USA

**Keywords:** Depression, Men who have sex with men, HIV prevention, PrEP, FTC/TDF, iPrEx

## Abstract

We conducted a longitudinal and cross-sectional analysis of depressive symptomology in iPrEx, a randomized, placebo-controlled trial of daily, oral FTC/TDF HIV pre-exposure prophylaxis (PrEP) in men and transgender women who have sex with men. Depression-related adverse events (AEs) were the most frequently reported severe or life-threatening AEs and were not associated with being randomized to the FTC/TDF arm (152 vs. 144 respectively OR 0.66 95 % CI 0.35–1.25). Center for Epidemiologic Studies Depression scale (CES-D) and a four questions suicidal ideation scale scores did not differ by arm. Participants reporting forced sex at anal sexual debut had higher CES-D scores (coeff: 3.23; 95 % CI 1.24–5.23) and were more likely to have suicidal ideation (OR 2.2; 95 % CI 1.09–4.26). CES-D scores were higher among people reporting non-condom receptive anal intercourse (ncRAI) (OR 1.46; 95 % CI 1.09–1.94). We recommend continuing PrEP during periods of depression in conjunction with provision of mental health services.

## Resumen

Llevamos a cabo un análisis logitudinal y transversal de la sintomatologia depresiva en iPrEx, del estudio de placebo controlado, con seleccion de brazo al azar. Involucra tratamiento oral diario de FTC/TDF que esta usado como quimoprofilaxis de pre-exposicion (PrEP) contra VIH en hombres y mujeres transgénero que tienen relaciones sexuales con hombres (MSM/TGW). Eventos relacionados a depresion (AEs) fueron reportados como los sintomas mas adversos o de riesgo de vida. Estos no fueron asociados con la seleccion al azar del brazo FTC/TDF (152 contra 144 respectivamente; OR: 0.66; 95 % CI: 0.35-1.25). La escala de depresion (CES-D), que proviene del centro de Estudios Epidemiológicos de Depresion y un “filtro” con cuatro preguntas para detectar suicidios contemplados no difirieron entre los brazos. Personas que reportaron ser forzados tener sexo, siendo su primer encuentro con sexo anal, tenian CES-D resultados mas altos (Coeff: 3.23; 95 % CI: 1.24 a 5.23) y fueron mas probable contemplar suicidio (OR 2.2; 95 % CI: 1.09 a 4.26). CES-D resultados fueron mas altos en personas reportando recibir penetracion anal sin condon (ncRAI) (OR: 1.46; 95 % CI: 1.09 a 1.94). Recomendamos continuar PrEP durante periodos de depresion, mientras simultaneamente recibir servicios de salud mental.

## Introduction

Depression is a highly prevalent condition that presents with psychological, behavioral, and physical symptoms that can be debilitating, interfere with self-care, influence social interactions, and lead to suicide [1]. Depression is the second leading cause of disability worldwide [2].

Studies conducted in the United States suggest that the prevalence of depression is substantially higher among men and transgender women who have sex with men (MSM/TGW) compared to men in the general population [3–5]. Population-based mental health surveys have found higher rates of major depression; anxiety; substance use; childhood emotional, sexual and physical mistreatment; and suicidal behaviors in individuals disclosing same-sex sexual behaviors or identifying as gay [3, 6–9]. In addition, MSM/TGW carry a disproportionate burden of the HIV epidemic, and contend with victimization, harassment, rejection and fear of rejection, and discrimination [10, 11]. Psychosocial health problems, including depression, increase risk for HIV acquisition via sexual practices [8, 12, 13], however the effects of depression on HIV acquisition may differ depending on depression severity. The EXPLORE study found a curvilinear relationship between the severity of depression and sexual risk taking in African–American MSM in the US, whereby moderately depressed individuals reported the most risky sexual behaviors compared to non-depressed or severely depressed individuals [14]. Childhood sexual abuse (CSA) in MSM/TGW has also been identified as a factor strongly associated with increased risk for HIV acquisition [15, 16]. History of CSA in MSM/TGW is associated with an increased likelihood of engaging in sexual behaviors that put them at risk for HIV infection [15].

Daily, oral Truvada^®^ (a coformulation of emtricitabine and tenofovir disoproxil fumarate, or FTC/TDF) is safe and effective for HIV pre-exposure prophylaxis (PrEP) [17–20]. The iPrEx study was a multinational, randomized, placebo-controlled trial that investigated the safety and efficacy of FTC/TDF PrEP in a cohort of MSM/TGW. Depression was one of the most frequently reported adverse events (AEs) in the study [18]. Previous studies of antiretroviral therapies (ARTs), including FTC and TDF in HIV-infected participants have reported high rates of depression-related AEs and raised concerns regarding the possible influence of these antiretroviral regimens on depression [21–23]. While subsequent studies have reported that efavirenz shows the strongest association with mental health diagnoses [24], the prescribing information and medication guide for FTC/TDF includes depression as a potential AE in HIV-infected individuals. Previous studies have not examined the effect of FTC/TDF on depression in absence of other ARTs or outside of the context of HIV infection. The iPrEx study presented a unique opportunity to look at the impact of FTC/TDF specifically on depression in a randomized manner in HIV-uninfected individuals.

As FTC/TDF for PrEP becomes increasingly popular, it is important to understand the role of depression in HIV acquisition risk and if PrEP affects depression. This analysis addresses these questions with three primary aims in the iPrEx cohort: (1) determine if FTC/TDF is associated with depression (compared to placebo), (2) describe the prevalence of and the risk factors for depression, and (3) examine how depression may be associated with sexual practices. These findings will be important in establishing the safety profile of FTC/TDF PrEP in HIV-uninfected MSM/TGW at risk for depression, and in understanding how depression may impact the risk of acquiring HIV in these populations.

## Methods

### Study Overview and Population

Details of the iPrEx clinical trial have been published elsewhere [18]. In brief, MSM/TGW were randomized to receive daily oral FTC/TDF or placebo plus a comprehensive package of prevention services, and were followed for HIV infection and safety outcomes. The trial was conducted in 9 cities around the world (San Francisco and Boston, United States; Cape Town, South Africa; Chang Mai, Thailand; Lima and Iquitos, Peru; Guayaquil, Ecuador; and Rio de Janeiro and São Paulo, Brazil).

The inclusion criteria were male at birth, at least 18 years old, HIV seronegative, and reporting sexual practices conferring risk for acquisition of HIV. Study visits occurred every 4 weeks and included rapid HIV antibody testing, adherence counseling, pill count, drug dispensation, and medical history. Computer-Assisted Self Interviews (CASI), administered at the screening and quarterly visits, collected information about demographics, drug and alcohol use, sexual practices, and behavioral risk factors. Information about sexual practices was also collected quarterly using an interviewer-administered questionnaire. Study details are available in the supplemental materials of the original report [18]. The iPrEx cohort enrolled 2,499 subjects; 83 % were enrolled in Latin America. The age of participants ranged from 18 to 67 years (mean 24 years), and the baseline characteristics of the two study arms (placebo and FTC/TDF) were similar. Participants provided written informed consent for all study procedures, which were conducted in accordance with the Institutional Review Board (IRB) guidelines at each participating institution.

### Measures

#### Depression and Suicide Adverse Events

Clinicians assessed AEs, including depression, every 4 weeks through the end of study follow-up. AEs that were grade 2 or higher, as determined by the DAIDS Table for Grading the Severity of Adult and Pediatric AEs Version 1.0, December, 2004 (clarification August 2009), all bone fractures, and all creatinine elevations were recorded on Case Report Forms (CRFs) and faxed to the study data management center (DF/Net Research Inc.). Staff trained in MedDRA coding followed the conventions outlined in the MedDRA Term Selection Points to Consider Document (release 3.8 based on MedDRA version 10.0) to code all reported AEs. The preferred MedDRA terms for depression AEs used in this study were “Depression”, “Depressed Mood”, “Depression suicidal”, ‘Major Depression”, “Suicidal Ideation”, “Suicide Attempt”, “Adjustment Disorder”, and “Adjustment Disorder with Mixed Anxiety and Depressed Mood”.

#### CES-D and Suicidal Ideation Screeners

Depressive symptoms were assessed at screening and at semi-annual visits using the widely used 20-item Center for Epidemiologic Studies Depression Scale (CES-D) validated in English, Spanish, Portuguese and Thai [25–30]. The CES-D is both valid and reliable as a screening tool for depressive symptomatology and provides an estimate of depressive symptom prevalence within a study population rather than a clinical diagnosis of depression [25, 26, 31]. In addition, since the CES-D has no items on suicide, a 4-item suicide ideation survey screener was also implemented. The 4 items of this screener were “I had thoughts about death”, “I thought about killing myself”, “I felt my family and friends would be better off if I were dead” and “I felt that I would kill myself if I knew a way”. An item endorsed as occurring “rarely or none of the time” was assigned a value of 1, “some or little of the time” was assigned 2, “occasionally or a moderate amount of the time” was assigned a 3 and “most or all of the time” was assigned a 4. The values for each of the items were computed (with a possible range between 4 and 16) [30, 32].

For aims 1 and 2 in which depression is the outcome of interest, CES-D scores (range 0–60) were analyzed continuously. For aim 3, in which depression is examined as a predictor of sexual practices, the CES-D scores were categorized for more meaningful interpretation of results. Previous studies of US MSM have categorized CES-D either dichotomously with CES-D scores ≥16 representing clinically significant depressive symptoms and CES-D scores <16 as absence of symptoms; or with 16–26 for moderate versus ≥27 for severe depressive symptoms [12, 33, 34]. However, given that the distribution of CES-D scores can vary across cultures, we chose to categorize CES-D scores by quartiles observed in our multi-cultural study population (CES-D ≤ 7; 7 < CES-D ≤ 12; 12 < CES-D ≤ 19; CES-D > 19) [12, 35]. For all analyses, suicide ideation scores were analyzed as a dichotomous variable indicating presence or absence of suicidal ideation, as defined by a score of 3 or greater on any individual item of the suicide ideation scale [30, 32].

At the time of the design and initiation of the iPrEx study in 2006–2007, depression screening instruments were not routinely used in prevention clinical trials. However, in July of 2009, motivated by the occurrence of 46 depression-related AEs in 41 individuals in addition to two suicide attempts, the study team incorporated the regular administration the CES-D and the suicide ideation screener to the study protocol. All participants who screened as having depressive symptoms or suicidal ideation received mental health assessments at the study site, and, in some cases, referrals for off-site psychiatric evaluations and follow-up. Not all sites were able to provide outside referrals because of the paucity of locally available mental health services. Implementation of these new study procedures occurred at each site depending on the local IRB review timelines. Given that most iPrEx participants enrolled in iPrEx prior to July 2009, CES-D scores from screening visits are missing for many participants. In cases of seroconversion, all CES-D collected after the seroconversion time points were excluded from our analyses.

#### FTC/TDF Drug Exposure

Drug levels were assessed using FTC and tenofovir (TFV) in blood plasma, and their active metabolites, FTC-triphosphate (FTC-TP) and TFV-diphosphate (TFV-DP) in viably cryopreserved peripheral blood mononuclear cells (PMBCs), by liquid chromatography/tandem mass spectrometry [36]. The drug detection variable of TFV-DP was categorized as: 0—below the level of quantitation; 1—drug detected ≤16 fmol/10^6^ cells; 2—drug detected >16 fmol/10^6^ cells using the previously established cut-off which is the intracellular concentration TFV-DP associated with a 90 % reduction in HIV acquisition relative to the placebo arm, in presence of FTC [36].

#### Predictors of Depressive Symptoms

Demographic covariates including age, race, ethnicity, alcohol consumption, and study site were collected from CRFs and CASI questionnaires at baseline. “*Trans*” identity was defined as self-identifying as either “trans” or “a woman” on the CASI questionnaire.

To explore the potential relationship between trauma and subsequent depression, we selected behavioral characteristics from the screening CASI that may reflect prior traumatic experiences. The characteristics analyzed were “fear of violence”, concern surrounding “acceptance amongst family members”, and history of “forced sex”. “Forced sex” was defined as having reported being “forced” or “manipulated” at time of anal sexual debut.

#### Sexual Practices

Participants were asked about their sexual practices over the past three months at screening and at each quarterly visit during an interview-administered questionnaire. Measures used for this analysis were: the total number of anal sex partners, the number of partners with whom participants reported non-condom receptive anal intercourse (ncRAI), and the number of partnerships when a condom was used. Reported sexual behavior was recoded to create a binary variable for any ncRAI.

### Statistical Analyses

All analyses were performed in STATA 13.1 (StataCorp, College Station, TX, USA).

#### Aim 1: Is FTC/TDF Associated with Depression in the iPrEx Cohort?

To minimize the potential bias caused by the late introduction of the CES-D screener, the primary analysis examined depression AEs by study arm. The number of individuals with depression AEs was compared in the FTC/TDF and placebo arm. A χ^2^ test was used to test for differences by arm. To compare the total number of depression AE events by arm, a mixed-level random effects logistic regression was performed that accounts for clustering by participant.

The secondary analysis looked at changes in depressive symptomology that may not have had a clinical manifestation by exploring the effect of randomization on CES-D scores. A generalized estimating equation model was used to look at the effect of randomization on CES-D scores throughout the course of study follow-up. Similarly, a mixed level logistic regression model was used to test the effect of randomization on the presence of suicidal ideation as measured by the suicidal screener.

To ensure that any effects of FTC/TDF on depression were not obscured by imperfect adherence to the study drug, an as-treated analysis was conducted using drug detection in PBMCs. Age, study site, and anti-depressant medication use were considered as potential confounders and included in the model.

#### Aim 2: Predictors of Depression in the iPrEx Cohort

Multilevel linear and logistic regressions clustering on study site and participant were used to examine the effect of demographics and childhood traumatic events on depressive symptoms and presence of suicidal ideation respectively.

#### Aim 3: Does Depression Influence Sexual Practices in the iPrEx Cohort?

Multivariate logistic regression was performed to explore associations between CES-D scores and reported ncRAI, the major risk factor for HIV acquisition in this cohort [37] while controlling for total number of partners, age, anti-depressant medication use and study site. The first available CES-D score and corresponding sexual behavior data were used.

### Missing Data and Sensitivity Analyses

#### Missing CES-D

In order to understand the potential effect of excluding those who never received a CES-D assessment as a result of delayed implementation, we compared baseline demographics and depression as a pre-existing condition between those who had a CES-D assessment and those that did not using multivariate logistic regression.

Additionally, to assess for potential selection bias, we explored whether or not depression or depressive symptoms show any evidence of increasing the rate of study drop out using a cox proportional hazards model.

#### Missing Drug Levels

Multiple imputation was used to fill in the randomly missing data for time points at which drug levels were not directly measured as described previously [36].

## Results

### Is FTC/TDF Associated with Depression in the iPrEx Cohort?

#### Depression AEs by Study Arm

Depression was the most frequently reported severe or life-threatening AE in the iPrEx trial [18]. There were a total of 296 depression-related AEs during follow-up. Suicidal ideation and attempts were considered grade 4 (life-threatening) AEs. Table 1 compares depression-related AEs between study arms. There were no significant differences between arms (active vs FTC/TDF) in number of participants reporting depression-related AEs (OR 0.81; 95 % CI 0.62–1.06), the absolute number of depression AEs (OR 0.66; 95 % CI 0.35–1.25), or grade 4 AEs (OR 0.5; 95 % CI 0.22–1.15).

**Table 1:** Depression-related clinical AEs, CES-D scores and number of participants who screened positive for suicidal ideation in iPrEx by study arm

	Placebo n (%)^e^	FTC/TDF n (%)	OR/Coeff^b^	95 % CI
Number of participants reporting depression-related AEs (grade 2 or higher)^a^	127 (10.18)	105 (8.37)	0.81	[0.62 to 1.06]
Number of depression-related AEs (grade 2 or higher)^a^	152 (51.35)	144 (48.65)	0.66	[0.35 to 1.25]
Number of grade 4 depression-related AEs^a^	53 (34.87)	40 (27.78)	0.5	[0.22 to 1.15]
CES-D score	12^**c**^	12^c^	0.2^b^	[−0.46 to 0.86]
Number of participants who screened positive for suicidal ideation^d^	250 (8.29)	230 (7.83)	0.9	[0.67 to 1.21]

*AE* Adverse Event*, CES*-*D* Center for Epidemiologic Studies Depression Scale, *FTC/TDF* emtricitabine tenofovir disoproxil fumarate, *OR* odds ratio, *95* *% CI* confidence interval, *Coeff* coefficient

^a^Clinical AEs, as defined by the NIH DAIDS Table for Grading the Severity of Adult and Pediatric AEs Version 1.0, December, 2004 (Clarification August 2009) http://rsc.tech-res.com/safetyandpharmacovigilance

^b^Linear regression coefficient

^c^Median CES-D score

^d^Positive for suicidal ideation is defined as a score ≥3 on any of the four questions in the suicide ideation screener

^e^Percentages refer to either % of total number participants, or % of total number of events

There were 36 suicide attempts or gestures during the study: 16 in the placebo arm, and 20 in the FTC/TDF arm (OR 1.26 95 % CI 0.65–2.43). There was no suicide. Attempts included ingestions of poisons or overdose of medications. Eight medication ingestions involved study drug, four of which occurred in the FTC/TDF arm and could have involved as few as two tablets or as many as 30 tablets, either alone or in combination with other medications. No clinical AEs were associated with these overdoses. The serum creatinine remained normal in all cases.

The protocol required that study drug be discontinued for grade 4 depression-related AEs. As any suicidal ideation is defined as a grade 4 AE, 75 depression-related AEs led to study drug discontinuation; of which 36 (49 %) were permanent and 38 (51 %) were temporary lasting 0.5–6.4 months. Three participants became HIV infected while being on one of these interruptions in study medication that were mandated by the protocol.

#### CES-D and Suicide Screening Scores by Study Arm

Overall, 2,131 of 2499 (85 %) enrolled participants completed at least one CES-D (1423 Spanish, 318 Portuguese, 267 English, and 107 Thai). CES-D completion was comparable in both study arms (1,076 placebo; 1055 FTC/TDF). Mean duration of study participation prior to completing a CES-D was 15 months (IQR 11–22 months). Most participants were between 18 and 24 years of age and identified as Hispanic/Latino ethnicity, with Spanish as their main language. Over 2/3 of the cohort had completed secondary education. For participants for whom a CES-D was available, the mean number of sex partners in the past 3 months at baseline was 18 partners, and 61 % of participants reported ncRAI in the past 3 months.

Table 1 depicts results of the generalized estimating equation and multilevel logistic regression indicating no difference in CES-D scores or suicidal ideation respectively by study arm. Median CES-D score was 12 and approximately 8 % of the cohort tested positive on the suicide ideation screener, regardless of study arm.

381 participants did not complete a CES-D, most of whom exited the study before the CES-D was implemented. The study baseline characteristics of participants for whom a CES-D was available were not substantially different than of those participants for whom a CES-D was unavailable with respect to race, ethnic group, education, site, alcohol use, and sexual practices at screening (Table 2). Mean age for participants who did not complete a CES-D was 1.6 years younger than those who completed at least one questionnaire (95 % CI 0.72–2.67), reflecting the younger average age of the Andean study sites participants.

**Table 2:** Demographic characteristics of iPrEx participants with at least one CES-D questionnaire (n = 2115) and without (n = 381)^a^

	CESD available	No CESD
	N = 2115	N = 381
Age (mean)	27.4	25.8
Race n (%)		
Black/African–American	180 (8.4)	34 (9.2)
White	372 (17.5)	61 (16.6)
Mixed/other	1458 (68.4)	263 (71.5)
Asian	121 (5.7)	10 (2.7)
Ethnic group n (%)		
Hispanic/Latino	1517 (71.2)	288 (78.3)
Non-Hispanic/Latino	614 (28.8)	80 (21.7)
Education n (%)		
Less than secondary	440 (20.6)	84 (22.8)
Completed secondary	743 (34.9)	141 (38.3)
Post-secondary	928 (43.5)	136 (37.0)
Region n (%)		
Brazil	318 (14.9)	52 (14.1)
Andes	1423 (66.8)	277 (75.3)
United States	209 (9.8)	18 (4.9)
South Africa	72 (3.4)	16 (4.3)
Thailand	109 (5.1)	5 (1.4)
No. of alcoholic drinks n (%)^b^		
0–4 per day	992 (46.5)	139 (36.5)
≥5 per day	772 (36.2)	159 (41.7)
Sexual practices at screening n (%)		
Number of partners in the past 12 months (mean)	16	21
Any non-condom receptive anal intercourse (ncRAI) in the past 12 weeks	1288 (60.4)	197 (53.5)
Any transactional sex in the past 24 weeks	848 (39.8)	179 (48.6)

^a^Some categories do not add up to 100 % due to no answers/missing responses. Three participants had questionnaires that were largely incomplete and therefore not used for this analysis

^b^On days subject drank in the past 4 weeks

For participants for whom we had CES-D scores available, higher CES-D scores were not associated with a greater hazard of dropping out of the study early (HR 0.92; 95 % CI 0.70–1.21). Additionally, the proportion having depression as a pre-existing condition was higher amongst those with at least one CES-D measurement than amongst those excluded from the analysis (6.29 vs. 2.45 % respectively; p = 0.11 after controlling for study site).

#### As-treated Analyses

The multilevel mixed effects linear regression using multiple imputed drug data showed no significant association between drug exposure and CES-D scores (Coeff. −0.23; 95 % CI −2.23 to 1.76) or suicidal ideation (OR 1.18; 95 % CI 0.58–2.41) after controlling for anti-depressant medication use, age, and site.

### Predictors of Depression

The mean CES-D score of the entire cohort was 14 (range 0–55). Table 3 presents the demographic characteristics and childhood risk factors for depressive symptomology. The multilevel mixed effects linear regression controlling for clustering on study site and study participant indicates that having reported forced sex at anal debut and identifying as TGW are significantly associated with higher CES-D scores on average. Younger participants also trended towards higher CES-D scores than the older participants.

**Table 3:** Predictors of depressive symptomology in 2,115 MSM/TGW iPrEx participants for which at least one CES-D was available^a,b^

	N^a^	Median CESD^b^	Coeff^c^	95 % CI^c^
Race				
Mixed/other	1458	12	ref.	
Black/African American	180	12	0.37	[−1.33, 2.07]
White	372	11	−0.88	[−2.16, 0.41]
Asian	121	12.5	−0.75	[−2.83, 1.34]
Hispanic ethnicity				
Not Hispanic/Latino	614	12	ref	
Hispanic/Latino	1517	12	−0.59	[−1.89, 0.71]
Age				
18–24	1049	12	1.25	[−0.07, 2.57]
25–29	439	12	0.95	[−0.47, 2.37]
30–39	404	12	1.06	[−0.35, 2.47]
≥40	239	12	ref.	
Forced Sex^d^				
No	2000	12	ref.	
Yes	83	15	3.23	[1.24, 5.23]
Alcohol^e^				
0–4 per day	982	12	ref.	
≥5 per day	770	12	0.36	[−0.43 to 1.17]
Education				
<Secondary School	440	13	ref.	
Secondary School	743	12	−0.29	[−1.37, 0.79]
Post-Secondary School	928	12	−0.4	[−145, 0.66]
*“Trans*” Identity^f^				
No	1863	12	ref.	
Yes	268	13	1.22	[0.51, 2.40]
Study Site (country)				
Praca Onze (BRA)	83	12		
USP (BRA)	66	12		
Fiocruz (BRA)	169	12		
INMENSA (PER)	759	13		
ACSA (PER)	413	12		
Equidad (ECU)	251	12		
San Francisco (USA)	131	9		
Boston (USA)	78	10.5		
Cape Town (ZFA)	72	12		
Chiang Mai (THA)	109	13		

Country codes: BRA Brazil, PER Peru, ECU Ecuador, USA United States, ZFA South Africa, THA Thailand

CES-D scores measured after seroconversion were excluded from analysis

Multivariate analyses show that reporting forced sex at anal debut and reporting “*trans”* identity were the only baseline characteristics significantly associated with an increase in CES-D scores when controlling for all other covariates

*AE* Adverse Event*, CES*-*D* Center for Epidemiologic Studies Depression Scale, *OR* Odds Ratio, *95* *% CI* confidence interval, *Coeff* coefficient

^a^Number of participants

^b^Median CES-D scores including all time points at which a CES-D was available (multiple observations per person)

^c^Results of multilevel mixed model with random effects for site and participant

^d^Forced sex is defined as having reported being “manipulated” or “forced” at the time of anal sex debut, as recorded in the CASI questionnaire

^e^On days when alcohol was used in the past 30 days prior to baseline characteristics were collected

^f^ *“Trans”* identity is defined as self-identifying as “trans” or “a woman” on the screening visit CASI questionnaire

When examining suicidal ideation as the outcome in a multivariate mixed level logistic regression, participants reporting forced sex also had 2.2 times the odds of having suicidal ideation than participants not reporting forced sex at anal debut (95 % CI 1.31–5.53). Additionally, black or African American participants had 2.15 times the odds of reporting suicidal ideation compared to participants of mixed/other race (95 % CI 1.09–4.26). No other variables were important predictors for suicidal ideation.

### Does Depression Influence Sexual Practices in the iPrEx Cohort?

Table 4 shows the effect of depressive symptomology on sexual behaviors. After adjustment for age, study site, anti-depressant medication use, and number of partners, participants in the highest quartile of CES-D scores had greater odds of reporting ncRAI in the past 3 months than those in the lowest quartile of CES-D. None of the other sexual exposures measures were associated with CES-D scores.

**Table 4:** CES-D scores and non-condom Receptive Anal Intercourse (ncRAI) in iPrEx participants

Odds of having reported ncRAI		
CESD Quartile	Odds ratio^a^	95 % CI
0–7	ref	
8–12	1.17	(0.88, 1.57)
13–19	1.21	(0.90, 1.61)
20 and above	1.46	(1.09, 1.94)
Wald test for linearity	p = 0.012	

Sexual practices data was collected at the same time the CES-D questionnaire was administered and reflects behavior over the prior 3 months. ncRAI is considered positive if a participant reports any instance of non-condom receptive anal intercourse during the interview-administered questionnaire

^a^Mixed level logistic regression using the first available CES-D score and corresponding reported sexual practices. Random effects for site were included. Analysis was controlled for number of partners, age of participant, and anti-depressant medication use

### Anti-depressant Medication Use

Use of anti-depressant medication at any time during study observation was reported in 211 (8.4 %) participants overall and there were no differences in anti-depressant medication use between study arms (8.6 % placebo vs 8.2 % FTC/TDF arm; Wilcoxon rank-sum z: 0.38 p = 0.71). Antidepressant use at any time during the study was reported by 50 % of participants with depression-related grade 2 + AEs.

## Discussion

During the iPrEx trial, daily oral FTC/TDF PrEP was not associated with depression; there were no significant differences in total number of depression-related AEs, CES-D scores, or suicidal ideation scores between study arms. As-treated analyses confirmed these findings, ruling out that similarity between groups could be attributable to lack of detectable drug exposure. Depression had been previously observed in trials of HIV-infected MSM receiving FTC/TDF in combination with other drugs for HIV treatment [21–23]. Antiretroviral medications used with FTC/TDF in these studies included efavirenz, which could have been responsible for these observations [38, 39]. In fact, recent evidence has linked efavirenz to neuropsychiatric effects and mental health diagnoses, including suicidal ideation, attempts or completion [24].

Though not related to oral FTC/TDF PrEP use, depression was one of the major AEs reported in iPrEx. Half of iPrEx participants who completed at least one CES-D had scores greater than or equal to 16 at some point over the course of the study, which is the cut-off considered to reflect clinically significant depressive symptoms in some settings [12, 26, 33]. In the iPrEx cohort, having been forced to have sex at anal sexual debut and ncRAI was associated with higher CES-D scores and there were trends toward higher scores among younger people and TGW. Our observations are consistent with previous findings that depressive symptoms are common in MSM/TGW, especially young people and those who identify as transgender women [10, 40]. MSM/TGW are at increased risk for social isolation, history of childhood abuse (including sexual abuse), and ongoing discrimination, which increase vulnerability to depression and suicide [6, 7, 9]. On the other hand, in this analysis, we did not see an association between alcohol use and CES-D scores. In-depth analyses of substance and alcohol use in iPrEx, and the subsequent iPrEx open label extension (OLE), as well as analyses of depression, other co-morbidities, and adherence are currently underway.

CSA is a traumatic early life event that adversely influences psychological and physical health in addition to socioeconomic well-being [41, 42]. CSA has been shown to be an important correlate of sexual risk-taking behaviors and HIV infection [15, 16]. CASI questions related to sexual debut may be relevant to assessing CSA; underage and forced sexual experience with an adult has been defined as CSA [15]. There is an association between having reported being “manipulated” or “forced” at anal sex debut and higher CES-D scores and increased likelihood of reporting suicidal ideation in the iPrEx cohort. The clinical significance of the observed 3-point average difference on CES-D scores is unclear. This relatively small difference seen on the CES-D may suggest that the influence of CSA on sexual behavior is mediated by other social and psychological variables, and not necessarily by depression [16, 43]. We did not see any association with any other questions pertaining to possible traumatic experiences; however these questions were not instruments specifically designed to screen for such events.

There were more frequent reports of ncRAI among those in the highest quartile of CES-D scores. This finding is consistent with previous studies showing depression to be associated with sexual practices that can increase acquisition of HIV infection [14, 33, 44]. Having more depressive symptoms may put the person at higher risk for acquiring HIV during sex, possibly because depression may undermine self-care, communication with partners, or perception of risk. Depression may interfere with discussions about condom use, and asserting a desire for protective use of condoms. In addition, depression may trigger use of substances, which could impair perceptions of risk or impulse control.

During iPrEx, three participants on the FTC/TDF arm seroconverted while on a protocol-required study drug interruption triggered by depression-related AEs. Several findings support a recommendation to continue PrEP during periods of depression. We saw no evidence that PrEP was less efficacious among participants having a CES-D score above 16 during the iPrEx trial (Mantel–Haenszel test for heterogeneity p = 0.92). There was no link between oral FTC/TDF PrEP and depression or depressive symptoms. Behavior linked to HIV risk was more commonly reported among people with depressive symptoms. Indeed, 3 HIV infections occurred during protocol mandated interruptions in study medication due to depression. The protocol requirement to stop PrEP during periods of depression was amended as the iPrEx trial transitioned from the randomized phase to OLE, based on the findings reported here. In the iPrEx OLE study, drug was only interrupted if participants reported suicidal ideation with a concrete plan.

Adherence to HIV treatment can be affected by depression [5, 45, 46]. Given the blinded placebo-controlled design of the iPrEx trial and the protocol requirement that study medication be stopped in participants with grade 4 depression AEs, the analysis of adherence to PrEP amongst depressed people during the randomized phase of iPrEx is difficult and subject to bias. However, analyses from iPrEx OLE examining the role of depression on adherence to open-label PrEP are currently underway.

Because iPrEx was conducted at 11 study sites across the globe, our analyses are somewhat limited by a wide variation in the cultural context, access to mental health services, and access to anti-depressant medications. Studies of depression in the Hispanic Health and Nutrition Examination Survey (HHANES) using CES-D scales suggest that results may need to be interpreted differently in Hispanic populations [47–49]. Though validated in Spanish, Thai, and Portuguese, CES-D scores and their clinical significance should be interpreted carefully cross-culturally. This limitation was partially addressed by analyzing the CES-D scores as continuous variables or divided into quartiles, and controlling analyses for random effects by study site.

Use of anti-depressants was low overall (8 % of entire cohort), being reported in only one half of people with depression-related AEs grade 2 or higher. Anti-depressant medication use varied dramatically by site (but not by study arm), with study sites in the USA reporting the highest proportions of psychotropic medication use. Anti-depressant medication use had no effect on our study findings. No drug interactions are expected between FTC/TDF and commonly used anti-depressant medications [50]. Individuals who are under care for depression should be evaluated for PrEP indications. In settings having a substantial prevalence of HIV infection, PrEP indications include inconsistent condom use outside of mutually monogamous relationship with a recently tested HIV-negative partner, any sexual partner who is HIV-positive or whose HIV status is unknown, any sexually transmitted infection by self-report or laboratory testing, use of post-exposure prophylaxis, or any sharing of needles for injection drug use [51]. Antidepressant therapy may improve PrEP adherence and uptake, as it has for antiretroviral therapy for HIV infection (reviewed in [52]).

A limitation of the current analysis is that plans to examine depression symptoms systematically using CES-D and the suicidal ideation screener were made after the trial was already underway, and were triggered in part by the high rates of depression-related AEs (depression AEs were evaluated throughout the entire study follow-up and therefore do not suffer from this same limitation). Although 15 % of participants never completed a CES-D, the available information this was evenly distributed across the FTC/TDF and placebo arms. Furthermore, CES-D scores, when available, did not predict earlier study drop out after controlling for age and study site. Additionally, when examining pre-existing conditions of depression that were recorded for all study participants, we see no difference in frequency of pre-existing conditions in participants who never received a CES-D versus those who did. These findings suggest that our finding that depressive symptoms were not associated with oral FTC/TDF PrEP is not likely to be substantially biased by missing CES-D scores.

Oral FTC/TDF PrEP does not affect depression incidence or severity and depressive symptoms are associated with sexual practices that increase risk for HIV acquisition. The additional protection conferred by PrEP could avert infections during these especially vulnerable times, while fostering engagement in health services. Adherence to PrEP and other safer sex recommendations may be especially challenging during periods of depression, so specific attention to promoting adherence during these periods is important. Given the high prevalence of depression and HIV incidence among MSM/TGW, especially young people, PrEP services should include screening and management of trauma, including CSA. PrEP programs may provide infrastructure for increasing access to mental health services, enabling synergistic diagnoses, and management of co-morbidities.
